# Identification of illegally dumped plastic waste in a highly polluted river in Indonesia using Sentinel-2 satellite imagery

**DOI:** 10.1038/s41598-023-32087-5

**Published:** 2023-03-28

**Authors:** Anjar Dimara Sakti, Emenda Sembiring, Pitri Rohayani, Kamal Nur Fauzan, Tania Septi Anggraini, Cokro Santoso, Vinka Aprilla Patricia, Kalingga Titon Nur Ihsan, Attar Hikmahtiar Ramadan, Sanjiwana Arjasakusuma, Danang Surya Candra

**Affiliations:** 1 Remote Sensing and Geographic Information Sciences Research Group, Faculty of Earth Sciences and Technology, Institut Teknologi Bandung, Bandung, 40132 Indonesia; 2 Air and Waste Management Research Group, Faculty of Civil and Environmental Engineering, Institut Teknologi Bandung, Bandung, 40132 Indonesia; 3 Center for Remote Sensing, Institut Teknologi Bandung, Bandung, 40132 Indonesia; 4 Geospatial Information Agency of Indonesia, Cibinong, 16911 Indonesia; 5 Department of Geographic Information Science, Faculty of Geography, Universitas Gadjah Mada, Yogyakarta, 55281 Indonesia; 6 National Research and Innovation Agency, Jakarta, 13220 Indonesia

**Keywords:** Environmental impact, Physical oceanography, Hydrology, Environmental impact, Sustainability, Freshwater ecology

## Abstract

Plastic waste monitoring technology based on Earth observation satellites is one approach that is currently under development in various studies. The complexity of land cover and the high human activity around rivers necessitate the development of studies that can improve the accuracy of monitoring plastic waste in river areas. This study aims to identify illegal dumping in a river area using the adjusted plastic index (API) and Sentinel-2 satellite imagery data. Rancamanyar River has been selected as the research area; it is one of the tributaries of Citarum Indonesia and is an open lotic-simple form, oxbow lake type river. Our study is the first attempt to construct an API and random forest machine learning using Sentinel-2 to identify the illegal dumping of plastic waste. The algorithm development integrated the plastic index algorithm with the normalized difference vegetation index (NDVI) and normalized buildup indices. For the validation process, the results of plastic waste image classification based on Pleiades satellite imagery and Unmanned Aerial Vehicle (UAV) photogrammetry was used. The validation results show that the API succeeded in improving the accuracy of identifying plastic waste, which gave a better correlation in the r-value and p-value by + 0.287014 and + 3.76 × 10^−26^ with Pleiades, and + 0.143131 and + 3.17 × 10^−10^ with UAV.

## Introduction

More than 3.5 billion people on Earth depend on aquatic ecosystems as food supply resources^1^. The sustainability of this marine ecosystem faces severe problems related to plastic waste pollution. According to Cressey et al.^2^ approximately 86 million of the 300 million metric tons of plastic waste generated by 192 countries is already in open water. This amount results from the accumulated waste of 1.4% to 2.5% of world plastic production, or approximately 8 million metric tons of new plastic that is not treated. If the current trend continues, marine plastic waste can double in ten years^3^. The main factor contributing to the large amount of plastic waste in the environment is the lack of a plastic material cycle for waste management^4,5^. Unsustainable human activities, accompanied by misaligned regulations, have triggered an increase in plastic consumption^6^. Low awareness and a lack of public knowledge regarding plastic waste management are the main factors in the unresolved plastic waste problem^7^. One of the habits that can damage river ecosystems is the direct tipping of waste by the community along the river^8,9^, which is being increasingly carried out during the rainy season^10^. Of the 80% of land waste that reaches the sea, 25% is plastic, which has escaped from waste infrastructure systems. This enters the river network and then becomes bound ocean material^11^; this data is based on a study conducted by Cozar et al.^12^ who estimated that 1.15–2.41 million tons of plastic have been dumped into rivers. In addition, the Helmholtz Center for Environmental Research^13^ discovered that rivers in India, such as the Ganges and Indus Rivers; Africa, such as the Nile and Niger rivers; China, such as the Yangtze, Yellow, Haihe, and Mekong Rivers; and Indonesia, such as the Brantas, Solo, Serayu, Progo, and Citarum Rivers, transport the majority of the plastic waste found in the oceans. This large volume of pollution causes plastic waste pollution in rivers and is categorized as “heavily damaging pollution”, which has a massive impact on the environment^14^. One impact of poor plastic waste management is the emergence of illegal dumping, which damages the river network ecosystem and directly affects poor living patterns in residential areas^15^.

Efforts to improve the monitoring system for the identification of plastic waste must be made to increase the accuracy of the collection rate calculation. Efforts to identify untreated plastic waste can be made through a geospatial technology approach either directly or indirectly. Various platforms that can be used by utilizing a geospatial approach include direct monitoring efforts through citizen science^16^ as well as spatial modeling of the results of the integration of land cover and socioeconomic data^17,18^, in which an integration process in one framework is required as a reference in the planning, decision-making process, decisions, and evaluations^19^. In addition, the use of remote sensing data based on aerial photos using an unmanned aerial vehicle (UAV) platform integrated with machine learning has increased work efficiency by automatically identifying plastic waste targets^20,21^. UAVs have been widely used in identifying plastic waste on beaches, as in the study by Martin et al.^22^ and rivers^23,24^. Wolf et al.^25^ developed a CNN tile-wise classification method to detect plastic litter in rivers and performed debris classification and quantification. This study is a development of previous machine learning studies, such as the study by Martin et al.^22^ using the random forest algorithm, Goncalves et al. using random forest four-color spaces, and Fallati et al.^24^ using CNN pixel-wise classification to identify plastic waste on the beach. However, one of the important issues in the use of UAVs as tools for monitoring plastic waste is the affordability and low intensity of monitoring, which means that they cannot be utilized optimally for sustainable environmental management at the municipal and national levels^26^.

The development of surface monitoring technology for plastic waste based on Earth observation satellites has become a potential approach for conducting monitoring with a broader range of massive data. Martinez-Vicente et al.^27^ conducted an initial assessment to monitor marine plastic debris from space by utilizing multiple images of various wavelengths contained in Earth observation satellite sensors. Zhu et al.^28^ and Biermann et al.^29^ maximized the use of high-resolution satellites by focusing on near-infrared (NIR) spectral reflectance, which is sensitive in detecting plastics on a water surface. Moshtaghi et al.^30^ also explored the potential spectral reflectance of Visible and Near-Infrared (VNIR) and shortwave infrared (SWIR) for the analysis of marine microplastics. An experimental study by Tasseron et al.^31^ used hyperspectral imaging data. Finally, the study of Themistocleous^32^ proposed several well-established indices for water feature extraction to investigate plastic litter in the sea. The sensitivity analysis values method (SAV) was used to validate which index had the best capability of detecting plastic debris in water and indicated that the most optimal index to identify plastic litter was the plastic index (PI). However, as a source of entry of plastic waste and damage to ecosystems in river areas, the development of plastic waste monitoring studies based on open remote sensing data with medium spatial resolution, such as Sentinel-2, has not been widely implemented in river study areas. Gomez et al.^33^ used a multi-image segmentation architecture approach to detect plastic waste in a river area by using Sentinel-2 data. One of the obstacles to monitoring plastic waste in rivers is the narrow area that is commonly found and the complexity of land cover around the river body. Therefore, it is necessary to develop studies that can increase the accuracy of plastic waste detection in river areas.

This study aimed to identify illegal dumping in the river area using an adjusted algorithm from a previous study of the mistocleous in 2020^32^, namely, those obtained through the adjusted plastic index (API) using Sentinel-2 satellite imagery data. Three observational data sets, namely through in-situ (UAV Imagery) and ex situ (Sentinel-2 and Pleiades Imagery) stages, were used to validate the identification of illegal dumping using machine learning algorithms. This study focused on monitoring the accumulation of plastic waste without considering the river flow. The illegal dumping area in this study is assumed to have lasted for a long time so that a comparative approach can be used with other data sources with short time differences. The novelty of this study is that it is the first to carry out an adjustment process to the plastic index (PI) algorithm, which is expected to be applied in riverbank areas with land cover complexity and water quality that are different from those of open seawater. In addition, this study also attempted to integrate the role of other data sources, such as the high-resolution Pleiades observation satellite and UAV photogrammetry, to determine the correlation between API and the percentage of plastic waste area at a pixel size of 10 m in Sentinel-2. This study hopes that the process of identifying illegal dumping in riverbanks can be carried out more efficiently and broadly in the future, so that the problem of river plastic waste as an entry point for plastic waste in the sea can be handled more efficiently.

## Material and methods

### Area of study

**Figure 1:**
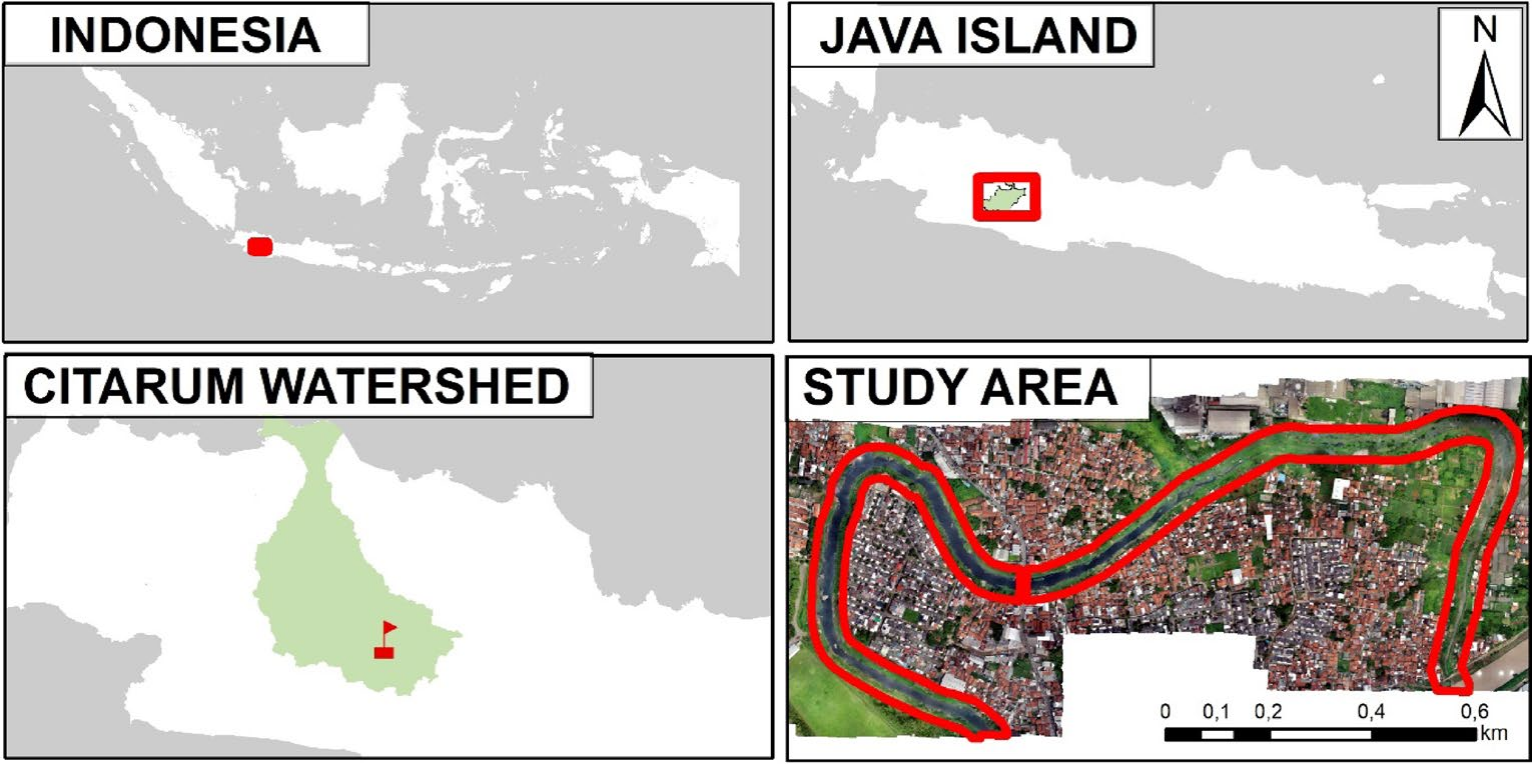
The location of open lotic-simple form type river as study area.

The study area as depicted in Fig. 1 is in Rancamanyar, Bandung Regency, West Java, Indonesia, along one of the Citarum tributaries. The Citarum River is largely responsible for its high level of waste pollution, and the Indonesian government has implemented a special programme to reduce pollution in the river network ^34^. The high level of waste pollution in the river body and on the riverbank, which is surrounded by residential areas with the potential for increased illegal dumping, became the primary reason this river was chosen for the research. Furthermore, the study's river type, an open lotic-simple form oxbow lake^35^ with slow water flow, makes it highly vulnerable to ecosystem damage. In addition, because of its small area coverage, the river area is also suitable for UAV flights, which is far from high-voltage air lines.

### Data used in this study

The data used in this study are listed in Table 1. The data processing phases are as follows: general, identification of illegal dumping points, and optical verification. A manual delineation process based on UAV data was used to obtain highly accurate river boundaries and illegal dumping areas. Sentinel-2 satellite data was used in the identification phase of illegal dumping areas, while high-resolution Pleiades images and orthophoto images from UAV shootings were used in the verification phase. In Identification of machine learning classificationa and error values used 843 sample points, with 70% as training data and 30% as testing data.

**Table 1:** Data used in this study.

Phase	Data	Format/resolution	Year data	Source	References
Base map process	River and illegal dumping delineation	Polygon	2020	In this study	–
Illegal dumping identification	Sentinel-2 MSI 2A	10 × 10 m	2017–2021, January 2019, Maret 2019 and December 2021	ESA	ESA, 2017^36^
Optical verification	Pleiades 1a/1b	0.5 × 0.5 m	February 2019	ESA	ESA, 2021^37^ LAPAN, 2021^38^
	UAV	0.05 × 0.05 m	December 2021	In this study	–

### Sentinel-2 satellite dataset

The optical satellite Sentinel-2 is a remote-sensing technology launched by the ESA as part of the European Commission’s Copernicus program on June 23, 2015. The satellite was equipped with a multispectral optoelectronic sensor for surveying. The spatial resolution ranged from 10 to 60 m in the visible, near-infrared (VNIR), and short-wave infrared (SWIR) spectral zones. Sentinel-2 has a temporal resolution of 5 days at the equator and 2–3 days at the midline (using combined constellation)^36^. The ESA Sentinel-2 MSI Level 2A optical satellite was used in this research as the main dataset for its use in identifying illegal dumping areas as hotspots. The Sentinel-2A imagery focused on three different time periods for waste detection: January 2019, March 2019, and December 2021. Alongside with monthly analysis from 2017 to 2022 to observe the pattern of changes in the overall of plastic index. Considering of Sentinel-2's higher temporal and spatial resolution compared to other open-source satellites with sensitive detection in the visible and NIR bands^32^, Sentinel-2 becomes suitable for this research topic.

### Pleiades satellite dataset

The plastic index that was developed in this research needs to be verified using satellite data with a higher spatial resolution than Sentinel-2, one of which is the Pleiades 1a/1b image with a spatial resolution of up to 0.5 m^37^. The Pleiades image was obtained from the LAPAN space map platform^38^, with the temporal data used from 2019 to 2020. Pleiades data consists of a panchromatic image with a resolution of 0.5 m, a pan-sharpened colour image with a resolution of 0.5 m, a multispectral image with four bands: red, green, blue, and NIR with a resolution of 2 m, and a combined image with a resolution of 0.5 m and 2 m. Pan-sharpened colour image (0.5 m) considered has the better spatial resolution hence was used to identify plastic waste in the river scale.

### Photogrammetry UAV dataset

Unmanned aerial vehicle (UAV) one of the remote sensing technologies that proficient to obtaining a picture of the earth’s surface at a very high spatial resolution. Photogrammetric image acquisition was performed with a DJI Phantom 4 Pro 2.0, a multirotor UAV equipped with a 20MP 1-inch RGB (red, green, and blue) CMOS camera sensor, and a mechanical shutter. The DJI Phantom 4 Pro features an optimized f2 from air and eight wide-angle lenses with a 24 mm equivalent focal length. The resulting camera resolution was 4096 × 2160 px and the ISO values ranged from 100 to 3200. This UAV has a GPS/GLONASS positioning system combined with a barometer and an Inertial Measurement Unit (IMU), which results in a hovering accuracy value of ± 0.5 m vertically and ± 1.5 m horizontally. Additionally, a lower vision system (DVS) was integrated with an accuracy of ± 0.1 m vertically and ± 0.3 m horizontally. The UAV flew at an altitude of 75 m with a theoretically generated ground sampling distance value of 2.05 cm. The GSD value was suitable for obtaining qualitative insights from plastic waste hotspots on riverbanks^10^. By flying high, the condition of the tributaries in its entirety could targeted, and waste hotspot points were obtained in a single tributary flow. The UAV analysis acquired a total area of 70 ha, which was divided into three flying missions. Each flying mission had a sidelap, overlap value of 80%, and flying speed of 9 m/s. Each flying block required 15 min to fly. The three missions produced 1200 photos, which were then processed to create a digital elevation model and orthophoto data.

### Methodology

**Figure 2:**
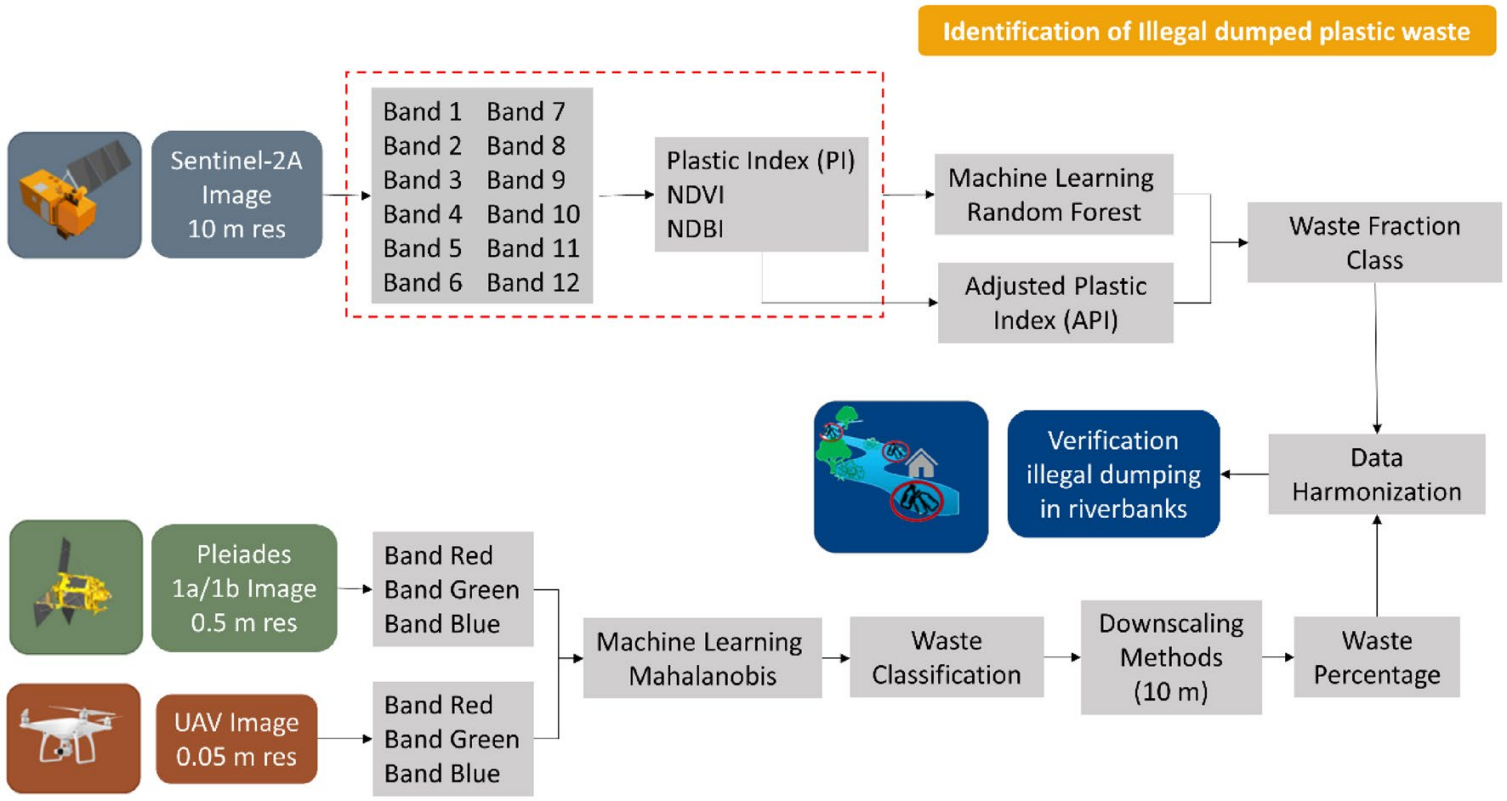
General framework employed in this study.

Three image datasets were used in this study: Sentinel-2A, Pleiades 1a/1b, and UAV imagery. Sentinel-2A is the primary data source for the Adjusted Plastic Index (API) in the river area, converting information from 12 band channels into three indices used in API calculations: Plastic Index (PI), Normalized Difference Vegetation Index (NDVI), and Normalized Difference Built-up Index (NDBI). The remaining two high-resolution images were used to validate the Sentinel-2A analysis results by calculating the waste percentage at a scale of 10 m. Subsequently, two machine learning methods were used to classify waste in the river area. Random Forest was used on Sentinel-2A imagery, while Mahalanobis was used on Pleiades and UAV imagery to perform waste classification, the results of which will be harmonized for the calculation results verification process. The general framework shown in Fig. 2 describes the flow of the method used in this study.

### Machine learning: random forest

Machine Learning (ML) Random Forest (RF) method were used for land cover and illegal dumping identification using supervised classes from Sentinel-2A imagery. RF is an ensemble learning method that can be used for either a categorical response variable named “Classification” or a continuous response, referred to as “Regression.” From a computational standpoint, RF is versatile because it naturally handles both regression and multiclass, is relatively fast to train and predict, and can easily be implemented in parallel^39^. An RF is a tree-based ensemble, with each tree depending on a collection of random variables. an RF uses trees *h*_*j*_ (*X*,*θj*) as the base learners. For the training data, *D* = {(*x*_*1*_,*y*_*1*_), . . . , (*x*_*N*_,*y*_*N*_)}, where *xi* = (*x*_*1*, *j*_, . . . ,* xi*,*p*)^*T*^ denotes the p predictors, ${y}_{1}$ denotes the response, and a particular realization θj is denoted *hj *(*x,θj,D*). To make a prediction at new pixel in Sentinel-2A imagery for classification, the RF algorithm is expressed in (Eq. 1)^40^.

1 $$f\left(x\right)={argmax}_{y}{\sum }_{j=1}^{J}I({h}_{j}\left(x\right)=y)$$

### Machine learning: Mahalanobis distance

For the land cover classification process, this research used the machine learning (ML) Mahalanobis distance (MD) method, which included identifying plastic waste as the primary target for both the Pleiades and UAV photogrammetry images. The MD method is a type of guided classification widely used for classifying remote sensing images^41^. The Mahalanobis distance method in the classification process was implemented by measuring the distance between two data samples in a multivariate space^42^. Mahalanobis distance classification method will find the nearest distance in a class. The application of multivariate in this method allows the classification process to be performed quickly and with high accuracy^43^. This application was beneficial, particularly in the classification process based on UAV image data with a very high spatial resolution over a large area and the target of plastic waste over a small area. Equation (2) shows the general equation for the classification of the Mahalanobis distance in an image^44^. Where d is the distance of Mahalanobis in a class, $X$ is the value data, $\overline{X }$ is the mean value of training data in class, $S$ is the covariance^44^. Each class will calculate for the mahalanobis distance. Pixel assigned to class when the d is the lowest from every class that have calculated^45^.

2 $$d= {\left[{(X-\overline{X })}^{T}{S}^{-1}(X-\overline{X })\right]}^\frac{1}{2}$$

### Development of adjusted plastic index

The adjusted plastic index (API) adopted the plastic index (PI) formula developed by Themistocleous et al.^32^ that shows in Eq. (3). The mistocleous's study put the PI formula and other index products to the test in order to detect floating plastic litter in open-water areas of the sea with a wide coverage, where the target is in the form of plastic bottles designed to be tied together and floated in the ocean. This study, on the other hand, went through several stages of adjustment to ensure that the PI calculation could be used in watersheds, particularly given the complexity of the conditions for the type of plastic waste mixed with other land covers. Land cover includes various types of vegetation, ground, and urban areas. Land cover corrections were made around the river, focusing on those that could detract from the PI results: Correction of vegetation and land cover.

Correction for vegetation is represented by The Normalized Difference Vegetation Index (NDVI) and land cover correction for land and buildings designated by the Modified Normalized Difference Built-up Index (MNDBI). This correction is necessary because the PI formula is highly sensitive to vegetated land, open land, and built-up areas. Equation (4) ^46^ shows the NDVI algorithm: Eq. (5) ^47^ represents the MNDBI algorithm: NIR, RED, and SWIR are the image products of the near-infrared, red, and short-wave infrared wavelengths on the Sentinel-2 satellite. The stages of the adjustment process on the PI using Eqs. (6) and (7), where ${PI}_{1}$ is the first adjusted PI after correcting NDVI and ${PI}_{2}$ is the second adjusted PI after correction for NDVI and NDBI. The adjustment process using NDVI and MNDBI is only applied to index values greater than zero, which is expected to reduce noise due to vegetation, ground, and buildings. NDVI can detect vegetation by observing the range of NDVI values that are more than zero (NDVI > 0)^48^. In addition, MNDBI can also detect land and buildings by considering the range of the index values that are more than zero (MNDBI > 0)^49^.

3 $$PI=\frac{NIR}{(NIR+RED)}$$

4 $$NDVI=\frac{NIR-RED}{(NIR+RED}$$

5 $$MNDBI=\frac{SWIR-NIR}{(SWIR+NIR)}$$

6 $$IF \left(NDVI>0\right) : {PI}_{1}=PI-NDVI, ELSE :{PI}_{1}=PI$$

7 $$IF \left(MNDBI>0\right) : {PI}_{2}={PI}_{1}-MNDBI, ELSE :{PI}_{2}={PI}_{1}$$

This study used the Google Earth Engine (GEE) cloud platform^50^ to simplify the process of calculating index values using long-term Sentinel-2 images from 2017 to 2021. The development of adjusted plastic index formula was applied as a function to any Sentinel-2 10-day temporal resolution image by going through the cloud masking process in the study area using the library developed on the GEE platform. A linear regression model with the least-squares approach^50^ was used in this study to detect the adjusted plastic index value rate of change at a specific time when the offset and scale values of the revised PI changes were observed. The PI band is the dependent variable and the time band, in this case, is the independent variable. Equation (8) shows the least-squares equation, where $a$ is the y-intercept or the value of $y$ when $x$ is equal to 0 and $b$ is the slope or rate of change in $y$ for each change or increase in the value in the $x$-direction. Equations (9) and (10) show the calculations for $a$ and $b$, respectively:

8 $$Regression Line :y=a+bx$$

9 $$a=y-bx$$

10 $$b=r{S}_{r}/{S}_{x}$$

### Data harmonization process

To account for the different spatial resolutions of the Sentinel-2, Pleiades, and UAV image datasets, data harmonisation was performed using resample method. The target resolution was 10 m, which equate with the spatial resolution of the Sentinel-2 images. The fraction value of plastic waste at each 10 m pixel in each of the Pleiades and UAV image classification results was obtained using a statistical zone process. This data harmonization process was used for comparative analysis with the plastic index data calculated from the Sentinel-2 image to obtain a correlation strength value between the adjusted plastic index and the percentage of plastic waste in the river.

## Results

### Illegal dumping area delineation

**Figure 3:**
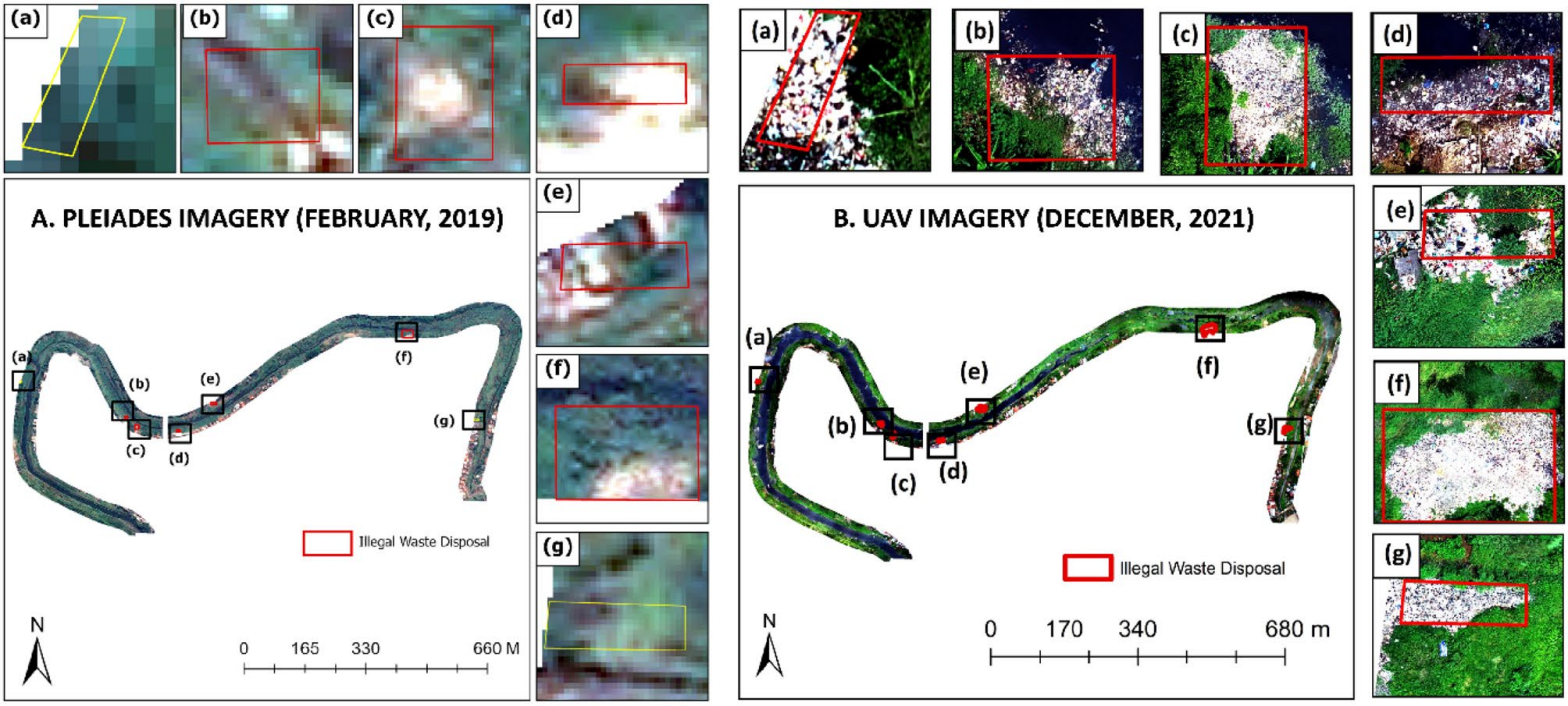
Seven illegal dumping areas were the target areas in this study: (**A**) Visualization results in the February 2019 with the Pleiades image, and (**B**) visualization results in December 2021 with the UAV photogrammetry images.

The illegal dumping area refers to the visualisation results from UAV image data that was manually digitized. The accuracy of the adjusted plastic index algorithm developed in this research was tested in this particular area. The river characteristics with extensive and long-term illegal dumping conditions have the advantage of processing multiple sources at different times because of illegal dumping tends to remain constant and its area grows every year. The selected illegal dumping area is also not affected by rising river water levels during high tide, because the location tends to be far from the coast. Therefore, the illegal dumping sites were areas that have a visual match between UAV and Pleiades images that could be compared with one another. Meanwhile, in the illegal dumping area, areas (a) and (g) visually show the results of the February Pleiades, which did not identify illegal dumping, whereas the UAV detected it. Figure 3A shows the distribution of illegal dumping areas used in this study in the actual color of the Pleiades image. Figure 3B shows the actual color of the UAV image.

### Waste classification on Sentinel 2 using machine learning

**Figure 4:**
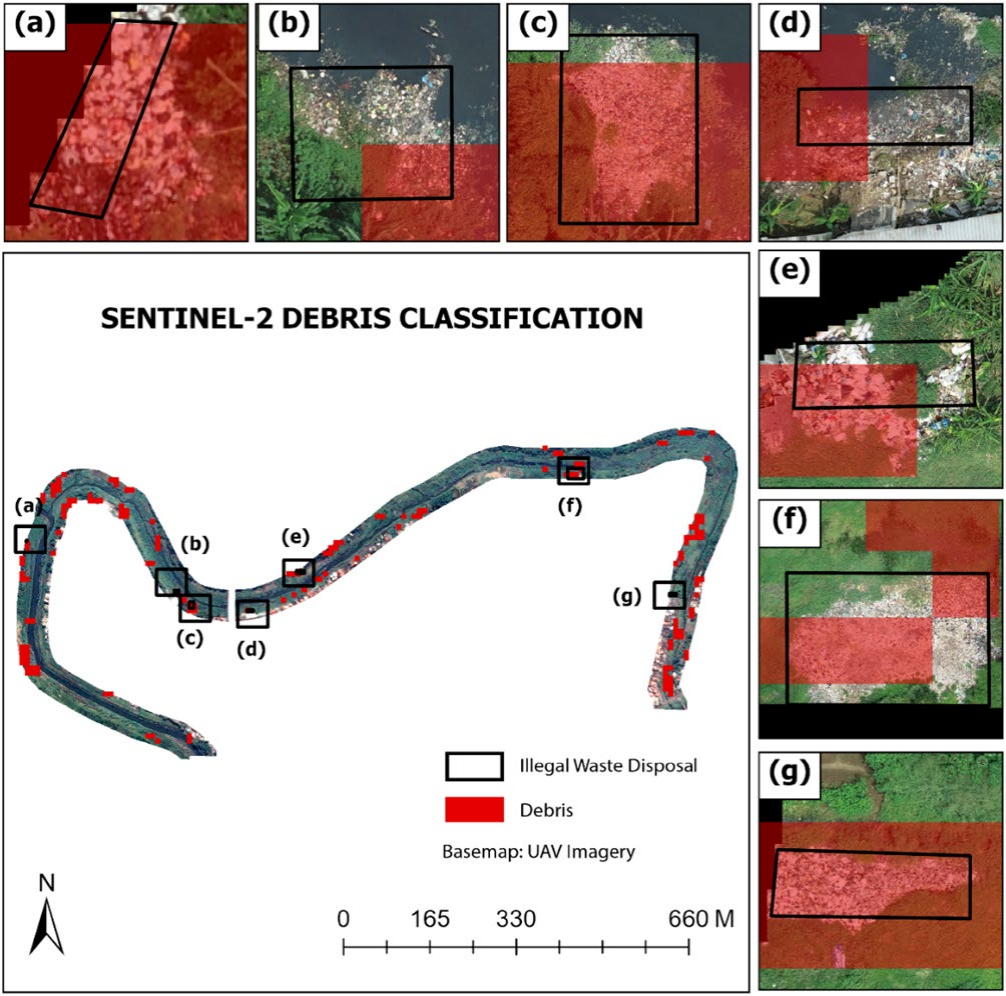
Sentinel 2A debris classification using NDVI, NDBI, PI Sentinel 2 + 12 Band Sentinel 2.

Figure 4 shows the results of the 12-Band Sentinel-2A classification using the random forest method, which is integrated with indexes—NDVI, NDBI, and PI. Using a dataset sourced from Sentinel-2A, the classification is based on five classes (buildings, debris, ground, water, and vegetation). The random forest algorithm is utilised to distinguish debris from other classes and produced a spatial resolution of 10 m × 10 m plastic waste class using the sentinel-2A dataset as an input. In general, the findings show that illegal dumping areas include not only the classification of plastic waste class but also the ground and buildings. This is due to the fact that plastic waste, the ground, and buildings all appear almost identical on satellite imagery. As a result, when gathering plastic waste sample data, it can be classified as either building or ground. However, in general, the illegal dumping areas can still be identified using random forest classification, although it does not have a high accuracy level.

### Adjusted plastic index

**Figure 5:**
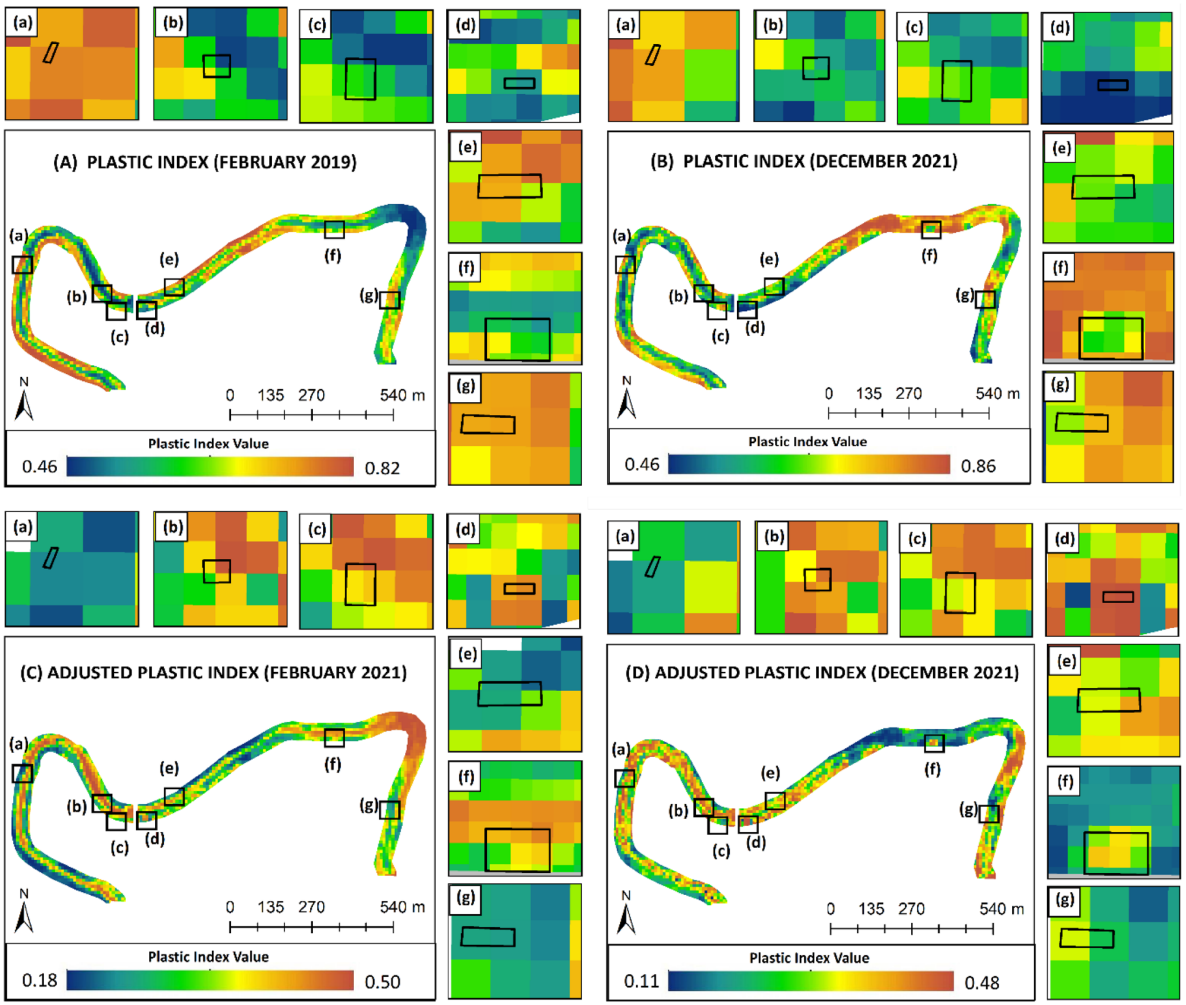
Result of: **(A)** PI in February 2019, **(B)** PI in December 2021, **(C)** API in February 2019, and **(D)** API in December 2021.

Figure 5 shows plastic index (PI) and adjusted plastic index (API) from the Sentinel-2 data. Figure 5A,B show the results of the PI products in February 2019 and January 2021, while Fig. 5C,D show the results of the API. There is a significant change in API compared to PI in February 2019 and December 2021. It can be seen that the distribution index of the highest PI was located on the river bend area in the API images. This result is consistent with how the process occurs of plastic waste being inhibited at river bends when meandering occurs. Furthermore, the area with the highest API is seen around the big bridge and resident’s settlements, which generally contain plastic waste originating from human activities living along the river. A comparison of the API in the seven target areas for illegal dumping shows a higher value than the PI. This result indicates that the adjustment process in this study can better represent the presence of plastic waste, particularly in river areas. The API images show the opposite impact of the high tendency in PI in the case of illegal dumping in areas (a) and (g). This is most likely due to a condition in which the February Pleiades data revealed that illegal dumping locations were not visible and even did not exist, in contrast to conditions in December 2021, which revealed that there were illegal dumping sites with a narrow area. This identified that the area with a high index value on the PI Algorithm shows an overestimated result, which has been successfully resolved through the adjustment stage. When comparing the API from February 2019 to December 2021, in general, the distribution of plastic waste followed the same pattern, especially in the illegal dumping area. Some locations have the most significant differences, which can be seen in the bend between the illegal dumping points f and g. This difference is caused by the location of the study, which is not an illegal dumping site, implying that the process of moving waste occurs at a low speed but is dynamic.

### Monthly analysis of the long-term adjusted plastic index

**Figure 6:**
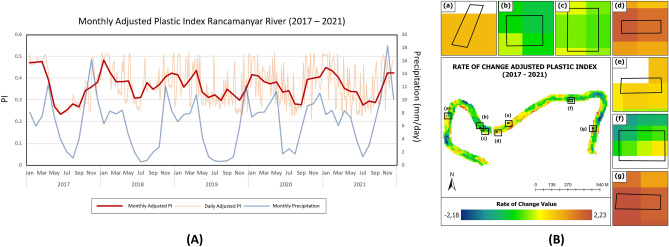
**(A)** Monthly and 5 days graph of the adjusted plastic index in all river study areas from 2017 to 2021 and **(B)** rate of change from the 2017–2021 adjusted plastic index data series.

Figure 6A shows the time-series results for the temporal API (every five days and monthly) values from 2017 to 2021. The average plastic index value fluctuated every month with an average range of values; the lowest was 0.23 in June 2017, and the highest was in January 2018 at 0.48. On the monthly chart, an increasing pattern can be seen every year from November to January, with the highest value always occurring in January during a period of five years. Meanwhile, the value of the plastic index decreased from June to September. This indicates that seasonal factors can influence the adjusted plastic index value. Figure 6A shows the pattern of change in the adjusted plastic index value compared to the average value of precipitation in the study area. Precipitation data were obtained from the Climate Hazards Group Infrared Precipitation with Station Data (CHIRPS V2)^51^. The rainy season lasted from October to May. The peak was from January to February, when the rainfall value reached 340 mm. There were 27 rainy days, and solar irradiation in the Bandung area was only approximately 30–40%. While the dry season occurs from June to August, when the amount of rainfall only ranges from 40 to 60 mm, the number of rainy days is nine, and the sunlight reaches 80%. From the data and information, it can be concluded that the plastic index value tends to increase during the rainy season and vice versa. The index value decreases during the dry season. This is because in the rainy season, the river discharge will increase and have the potential to carry more garbage, whereas in the dry season, the river water discharge will be lower, and the waste will be difficult to wash away. Figure 6B shows the rate of change of the plastic index value that occurred in 2017–2021, with positive changes indicated by red and negative changes indicated by blue and green-yellow, indicating that there was no significant change or index value relatively constant. The figure shows that only illegal dumping areas b, c, and f have a reasonably consistent index for five years, whereas a, d, e, and g have positive changes, which means that the index value is increasing in that period. The highest rate of change among the seven illegal dumping locations was observed at Site g, which had a rate of 2.21.

### Waste classification on Pleiades and UAV results

**Figure 7:**
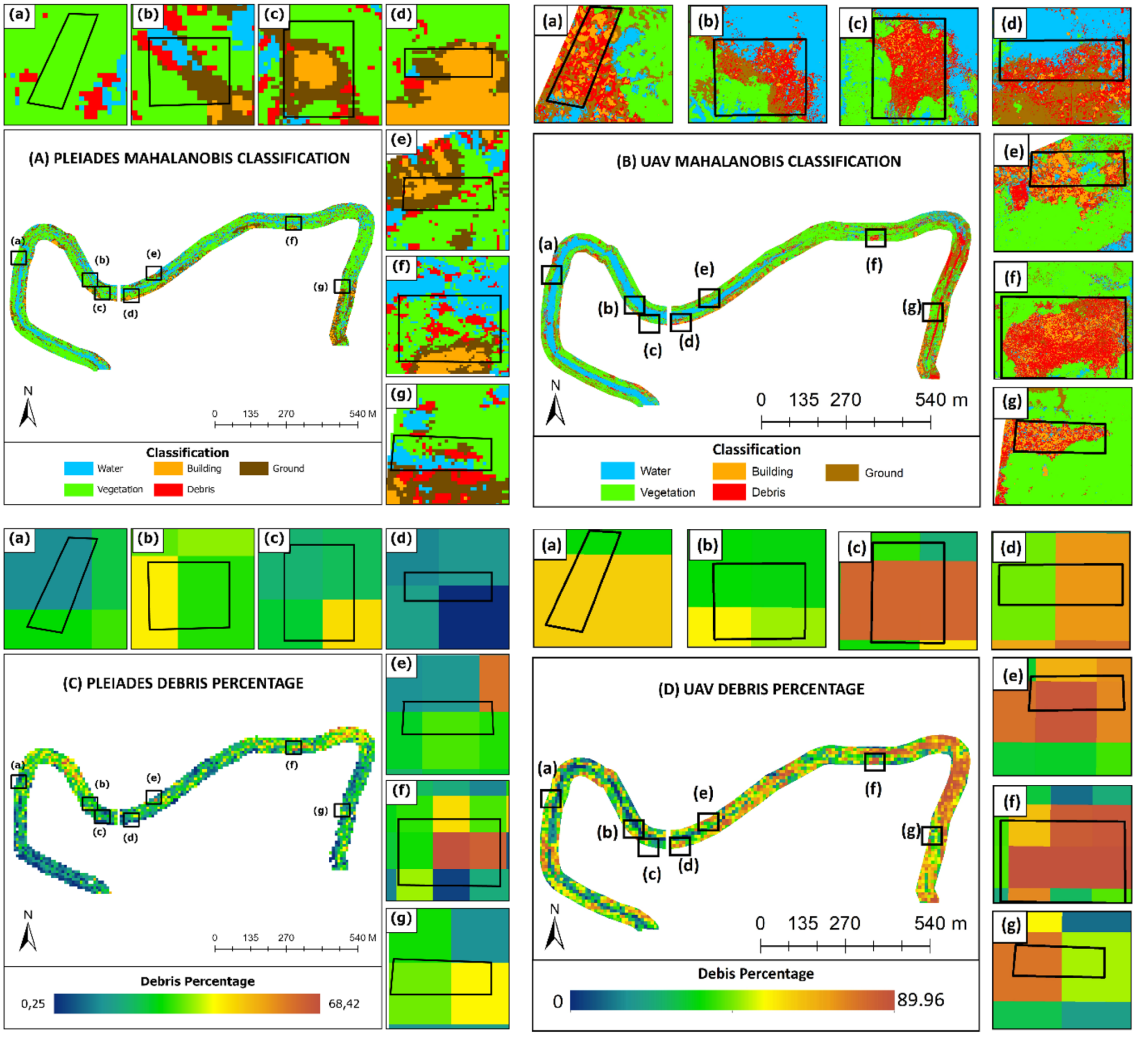
Results of the classification analysis of the five target classes using the machine learning Mahalanobis method on **(A)** Pleiades imagery and **(B)** UAV photogrammetry, as well as the results of the resampling to a pixel size of 10 × 10 and the percentage of debris on **(C)** Pleiades imagery and **(D)** UAV debris.

The classification results for the Pleiades and UAV images using the Mahalanobis method are shown in Fig. 7A,B. Five classes (water, building, ground, vegetation, and debris) were processed and applied to two Pleiades and UAV photogrammetry datasets. Specifically, for the target debris class, the spatial resolution was resampled to a size of 10 × 10 m with percentage units. Figure 7C,D depict the conversion of the percentage value of debris at a spatial resolution of 10 m from the Pleiades 0.5 m and the UAV 5 cm debris classes. In general, the two images show a pattern of plastic waste distribution that is nearly identical to the Mahalanobis classification of the Pleiades image, where the river bends and riverbanks had the highest percentage of waste. However, the distribution of the debris percentage value was 10 m × 10 m from the Pleiades image. The results from February 2019 show a smaller area and percentage range. The percentage range value in the Pleiades image has a value range of 0–70%, whereas the UAV image has a higher range of 0–80%. The high percentage value of up to 80% in UAV photogrammetry results after being resampled to a size of 10 m demonstrates the potential of Sentinel-2 data for identifying the amount of plastic waste, particularly in river water areas. The percentage of debris in the seven Mahalanobis classification areas in the Pleiades image generally showed a reasonably varied pattern. Illegal dumping areas, which should be dominated by plastic waste, have other class elements such as water, buildings, ground, and vegetation. The most significant distortions in the waste class were observed in the building and ground classes. This is because the waste, ground, and building classes have similar spectral visualizations, causing bias when taking training samples and classifying each method. Figure 7C,D show that the highest percentage of waste was 89.96% with a pixel area of 10 × 10 m. The points of the garbage piles were dominated by a yellowish-green to red color. Overall, the Mahalanobis method is quite effective at detecting objects. However, distinguishing waste from buildings and land still remains to be challenging.

## Discussion

### Accuracy of Sentinel 2, Pleiades, and UAV image classification

**Figure 8:**
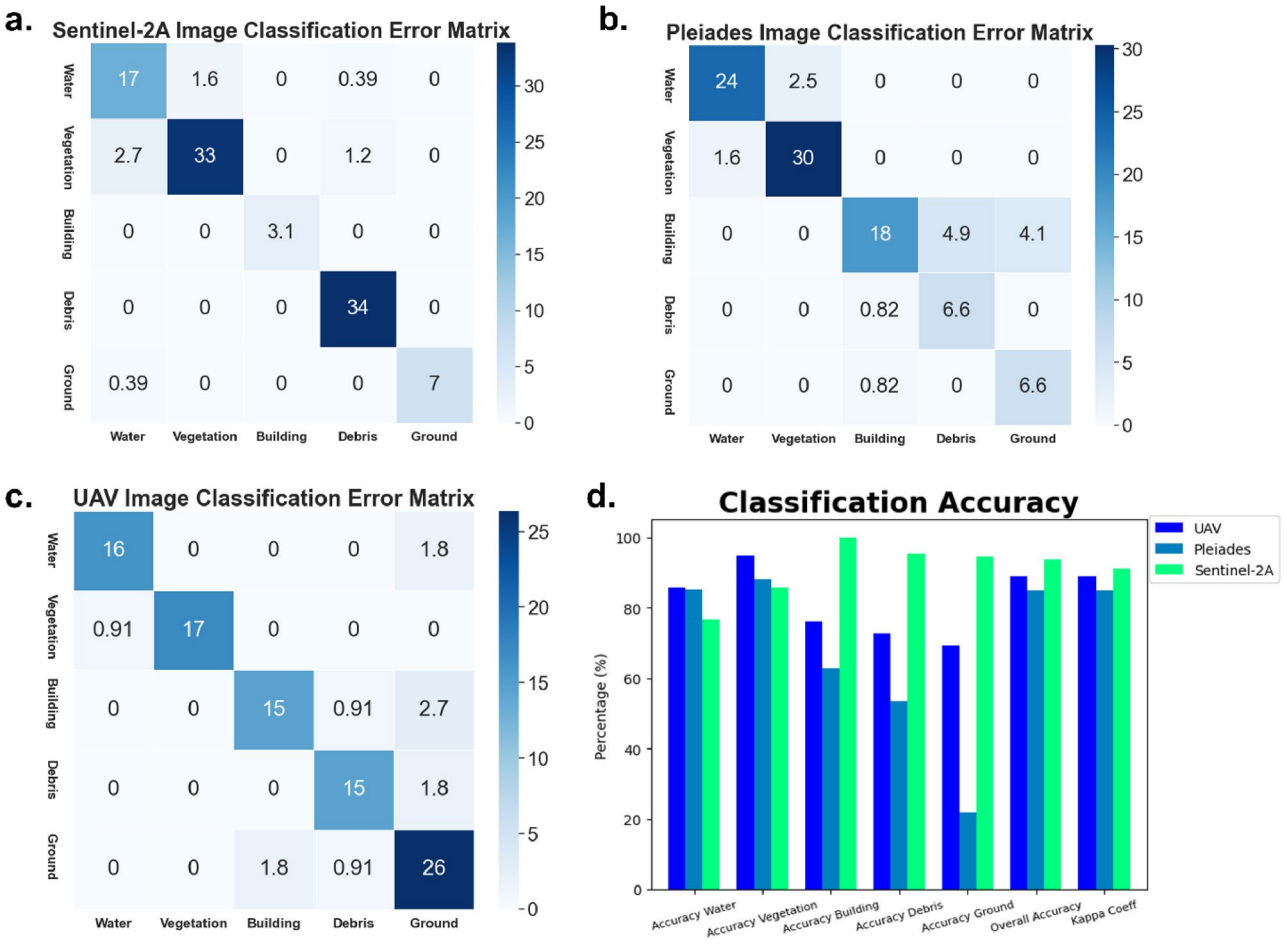
Results of the classification error matrix percentage on: **(a).** Sentinel-2A imagery, **(b)**. Pleiades imagery, **(c)** UAV photogrammetry and **(d)** classification accuracy percentage for the three approaches.

Figure 8 shows the statistical visualisation of the user accuracy percentage of image classification using machine learning. where (a) is the error matrix of the Sentinel-2A image, (b) Pleiades, (c) UAV, and (d) are the classification accuracy and kappa coefficient values of the three approaches used. Based on the accuracy results of the user, as shown in Fig. 8d, the accuracy for the class of debris from each image is Pleiades for 53.34%, UAV for 72.7%, and the highest is Sentinel-2A for 95.6%. Figures 8b,c depict similar debris classification and distribution patterns in Pleiades and UAV imagery, where waste objects were misclassified and detected as building and ground errors. This condition is due to the visual similarity of the three classes, which causes the results of waste classification to vary in an area. Whereas in Fig. 8a for the sentinel-2A image, the debris class classification is slightly affected by vegetation and water land cover, not ground or buildings. This is due to the difference in Sentinel-2's spatial resolution, where the pixels classified as ground and buildings are relatively few because their area is also not as large as vegetation and water land cover on the area. The error value is calculated using 843 sample points, which are divided into 70% training data and 30% testing data. The largest omission error on Sentinel-2A is water classification, which are 2.7% for vegetation and 0.39% for ground class. As for the commission error, it is 1.6% for vegetation and 0.39% for debris class. Whereas in the Pleiades imagery, the highest omission error is in the building class, with 0.82% for debris and the ground class. The commission error is 4.9% for debris and 4.1% for ground. As for UAV classification, it has the highest classification error in ground classification, with omission error values of 1.8% for water, 2.7% for buildings, and 1.8% for debris. Meanwhile, the commission error for UAV classification is 1.8% for debris and 0.91% for buildings.

### Comparison of adjusted plastic index models with plastic percentage

**Figure 9:**
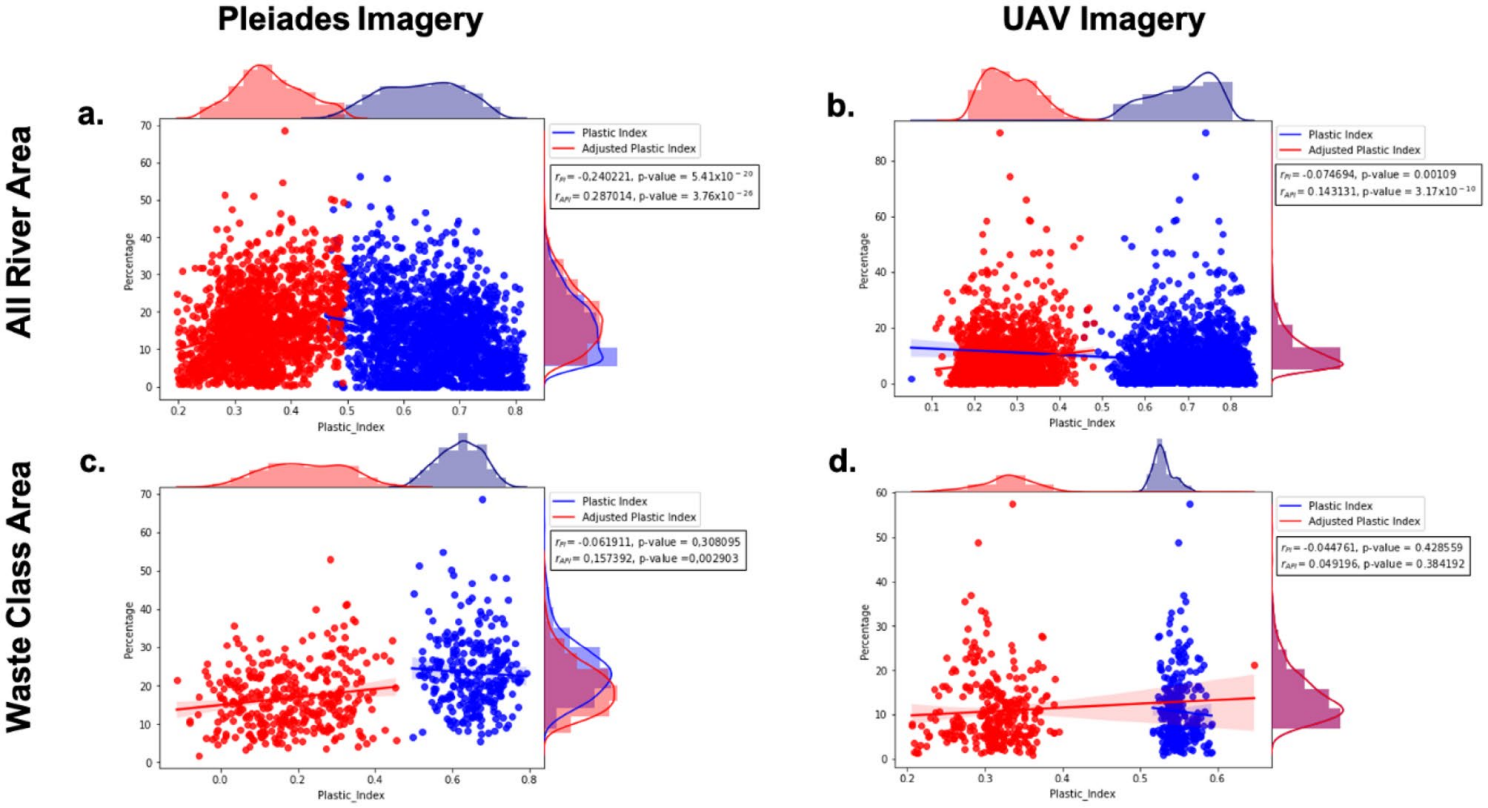
Scatterplot comparison between the percentage of plastic waste from Pleiades imagery and UAV imagery with plastic index (PI) and adjusted plastic index (API) of Sentinel-2: **(a)** comparison with Pleiades imagery for the entire river area, **(b)** comparison with UAV imagery in all river area, **(c)** comparison with Pleiades imagery in Sentinel-2 waste class area, **(d)** comparison with UAV imagery in Sentinel waste class area.

Referring to the goal of this research, which is to identify plastic waste in the river area using a modified PI formula from a previous Themistocleous study in 2020^32^, we compared the PI formula and adjusted PI in our study area. Figure 9a,b show the correlation between the percentage of the plastic index (PI) and adjusted plastic index (API) values from the Pleiades imagery in the river area and the waste class area from Sentinel-2. The percentage of plastic waste in the Pleiades imagery shows that the PI in February 2019 had a negative correlation in both areas, with an r value of −0.240221 for the whole river area and −0.061911 for the waste class area. In addition, the p-value of the river area was 5.41 × 10^−20^ and that of the waste class area was 0.308095. The correlation of the API improves with a positive trend, which decreases the figure of the r value by 0.287014 and the p-value by 3.76 × 10^−26^ to 0.157392 and 0.002903, respectively. This result is also consistent with the correlation between the percentage of plastic waste with PI and API in the UAV imagery. Figure 9c,d depict the results of this analysis, which show that after the adjustment, the correlation values of river (0.074694) and waste class area (−0.044761) increased to river (0.143131) and waste class area (0.049196), whereas the p-values of river (0.00109) and waste class area (0.428559) decreased to river (3.17 × 10^−10^) and waste class area (0.383192). These results indicate that the API improved the accuracy of plastic waste identification by increasing the r-value and decreasing the p-value. The improvement trend from the adjustment, showed insignificant growth. However, the API has improved the accuracy of identifying plastic waste, resulting in a higher correlation value (r-value) because it is positively correlated with the percentage of plastic waste. Overall, this study shows that API can improve the accuracy of the PI formula by Themistocleous, et al. (2020)^32^ in detecting plastic waste, particularly in river areas.

### Machine learning parameter relation index

**Figure 10:**
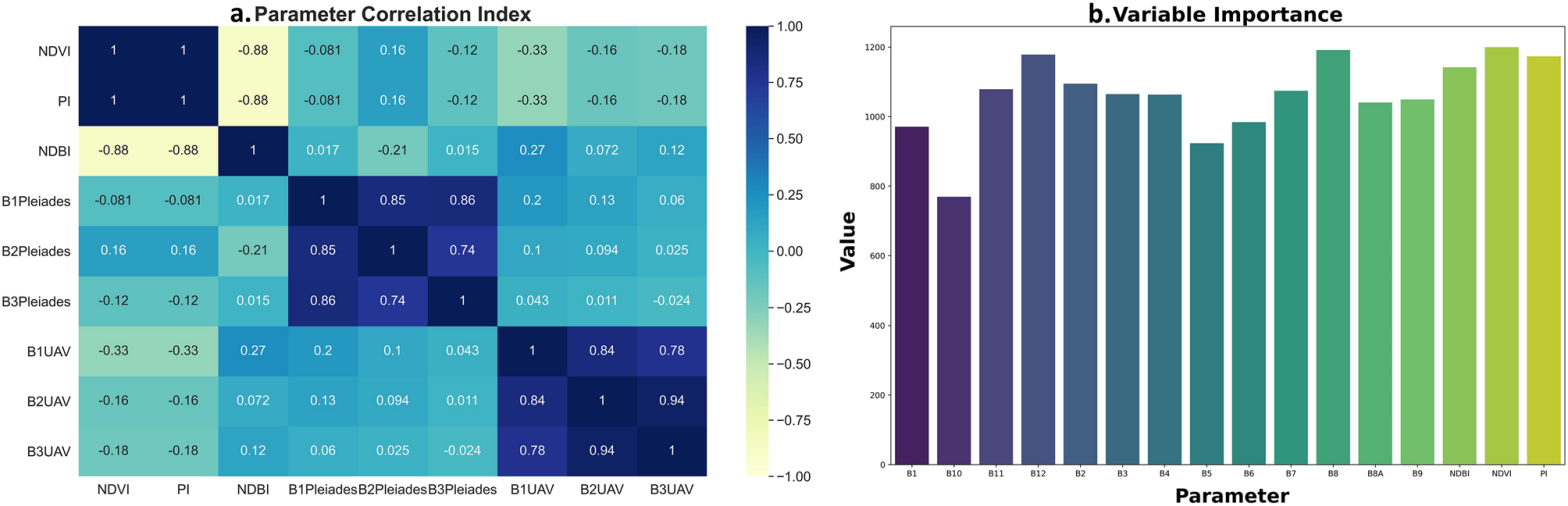
**(a).** Parameter corelation matrix index of Sentinel-2A, Pleiades, and UAV imagery, **(b)** value of variable contribution in product development of API.

The correspondence between the parameter index of Sentinel-2A satellite imagery with Pleiades Canal 1a/1b and the results of UAV photogrammetry shows in Fig. 10. The plastic index (PI) of Sentinel-2A has an inverse correlation with all UAV photogrammetric band channels, with the highest correlation value in the red channel at 0.33, green at 0.16, and blue at 0.18. whereas the PI with the channel in the Pleiades 1a/1b satellite image has a correlation and is inversely proportional to the red (0.08) and blue (0.12) channels, and is directly proportional to the green (0.16). This result is also aligned with the results in Fig. 10a, namely, the correspondence between the percentage of plastic waste in the UAV imagery and the Plastic Index (PI). In other words, the results of the parameter correlation of each index can be used to integrate data between Sentinel-2A, Pleiades 1a/1b, and UAV imagery to validate the identification of illegal waste dumping in the river. The 15 parameters used as predictor variables were analyzed to determine the level of influence of each variable on the developed API product (Fig. 10b). Based on the results of the variable contribution analysis, B2 and B6 were found to have the highest values. The other most important variables were NDVI, NDBI, and PI, all of which were related to the developed API model.

## Study limitation and future study direction

As an initial study for monitoring plastic waste in rivers based on Sentinel-2 satellite remote sensing data, this study had several limitations. For example, the target for plastic waste is still limited to the illegal dumping of a relatively large area of plastic waste under static conditions. In other words, this study was not able to identify a floating plastic debris target with small size and dynamic movement. Furthermore, the specified types of waste were assumed to be homogeneous, with the target being plastic waste. Meanwhile, under field conditions, there are various other types of mixed wastes. One of the issues in applying the supporting index parameters used in the plastic waste index adjustment process is the application of only two parameters: NDVI and NDBI. This condition is one of the reasons for overestimation of identification results. Another condition in this study is that the time of observation of the three remote sensing sensors was not accurately matched for Sentinel-2A, Pleiades, and UAV data, which is one of the reasons why the comparative analysis value has not been maximized when the time difference has the potential to change the conditions on the surface. This study identified that one of the keys to the successful identification of plastic waste is the open lotic-oxbow lake river, which allows it to target plastic waste within a large area and is statically immobile. In future studies, it will be necessary to investigate the response to the development of this plastic index adjustment algorithm for other river types. Another study that could be developed, especially by applying this adjustment approach to areas with different river types, is to identify objects that can increase the errors in the plastic waste index estimation process. For example, some types of rivers have the characteristics of very large riverbank housing activities, transportation activities that cause wave patterns, distribution of raised rocks, and water quality characteristics such as sedimentation, vines, and other water quality parameters that require further investigation. As a result of these characteristics, several other index correction alternatives, such as the normalized difference water index (NDWI)^51^, turbidity index^31^, and bare soil index (BSI)^52^, may be able to eliminate the effect of turbidity in water, soil, and others. Further research could improve in situ acquisition similarity so that multisensor observations can be performed with the same temporal resolution by predicting the temporal resolution of each sensor. The application of machine learning and deep learning to Sentinel-2 satellite imagery, in particular, is expected to play a strategic role not only in identification but also in waste counting and classification.

## Conclusion

This study aimed to identify illegal dumping in river areas using an adjusted plastic index (API) algorithm on Sentinel-2 satellite imagery data integrating with machine learning method. The results were obtained by first performing the classification process on Pleiades and UAV images using the Mahalanobis method on five target classes: water, building, ground, vegetation, and debris. Based on the calculation results of the Pleiades image classification error matrix, the values with the highest mapping accuracy were vegetation and water, reaching 88.10% and 85.29%, respectively. The classifications with relatively moderate accuracy were buildings, debris, and ground, which reached only 62.86%, 53.33%, and 21.79%, respectively. This result is due to the spectral similarity between debris, soil, and buildings, causing a bias in the study area, as previously described. Based on the classification of debris classes, the data were harmonized by performing a downscaling process to calculate the presentation value of plastic waste at a resolution of 10 m, according to Sentinel-2. The percentage of debris in the Pleiades image has a range of values between 0 and 70%, while the UAV image has a higher range, which 0–80%. The machine learning results are then used to determine the relationship between the percentage of plastic waste and the API model. The validation results show that the API has succeeded in improving the accuracy of PI formula, which gives a better correlation in r-value and p-value in the whole river area by 0.287014 and 3.76 × 10^−26^ with Pleiades, 0.143131 and 3.17 × 10^−10^ with UAV. To conclude, the API generally showed an increase in the index value of the seven illegal dumping targets compared to the results of the PI formula. Furthermore, this research has several limitations and requires further research, particularly in its application in different types of river areas and those with more diverse land cover. With the growing number of API formula implementations in various types of rivers, it is possible to make adjustments using other indices such as the turbidity index and the bare soil index. The use of machine learning and deep learning on Sentinel-2 could then be used in a future study to calculate the amount of plastic waste in water.

## Data Availability

The datasets generated and/or analyzed during the current study are available upon reasonable request to the corresponding author. Additionally, a platform for visualization of the data is available through the Google Earth Engine Application at https://gisact.org/geoplatform/plastic-river-indonesia/.
